# Vascular Inflammation Is a Risk Factor Associated with Brain Atrophy and Disease Severity in Parkinson's Disease: A Case-Control Study

**DOI:** 10.1155/2020/2591248

**Published:** 2020-07-14

**Authors:** Chiun-Chieh Yu, Hsiu-Ling Chen, Meng-Hsiang Chen, Cheng-Hsien Lu, Nai-Wen Tsai, Chih-Cheng Huang, Yung-Yee Chang, Shau-Hsuan Li, Yueh-Sheng Chen, Pi-Ling Chiang, Wei-Che Lin

**Affiliations:** ^1^Department of Diagnostic Radiology, Kaohsiung Chang Gung Memorial Hospital, Kaohsiung, Taiwan; ^2^Department of Neurology, Kaohsiung Chang Gung Memorial Hospital, Kaohsiung, Taiwan; ^3^Department of Internal Medicine, Kaohsiung Chang Gung Memorial Hospital, Kaohsiung, Taiwan

## Abstract

**Introduction:**

Systemic inflammation with elevated oxidative stress causing neuroinflammation is considered a major factor in the pathogenesis of Parkinson's disease (PD). The interface between systemic circulation and the brain parenchyma is the blood-brain barrier (BBB), which also plays a role in maintaining neurovascular homeostasis. Vascular cell adhesion molecule-1 (VCAM-1) and microRNAs (miRNAs) regulate brain vessel endothelial function, neoangiogenesis, and, in turn, neuronal homeostasis regulation, such that their dysregulation can result in neurodegeneration, such as gray matter atrophy, in PD.

**Objective:**

Our aim was to evaluate the associations among specific levels of gray matter atrophy, peripheral vascular adhesion molecules, miRNAs, and clinical disease severity in order to achieve a clearer understanding of PD pathogenesis.

**Methods:**

Blood samples were collected from 33 patients with PD and 27 healthy volunteers, and the levels of VCAM-1 and several miRNAs in those samples were measured. Voxel-based morphometry (VBM) analysis was performed using 3 T magnetic resonance imaging (MRI) and SPM (Statistical Parametric Mapping software program). The associations among the vascular parameter, miRNAs, gray matter volume, and clinical disease severity measurements were evaluated by partial correlation analysis.

**Results:**

The levels of VCAM-1, miRNA-22, and miRNA-29a expression were significantly elevated in the PD patients. The gray matter volume atrophy in the left parahippocampus, bilateral posterior cingulate gyrus, fusiform gyrus, left temporal gyrus, and cerebellum was significantly correlated with increased clinical disease severity, the upregulation of miRNA levels, and increased vascular inflammation.

**Conclusion:**

Patients with PD seem to have abnormal levels of vascular inflammatory markers and miRNAs in the peripheral circulation, and these levels are correlated with specific brain volume changes. This study reinforces the associations among peripheral inflammation, the BBB interface, and gray matter atrophy in PD and further demonstrates that BBB dysfunction with neurovascular impairment may play an important role in PD progression.

## 1. Introduction

Parkinson's disease (PD) has been characterized as a progressive neurological disorder without any available cure or preventative treatment [1, 2]. A diagnosis of PD can be confirmed by a postmortem examination of the substantia nigra for the loss of pigmented neurons and the presence of Lewy bodies (amyloid filaments) in the remaining neurons [3]. How does this neuron damage/loss happen in the brain? Recent studies have suggested that neuroinflammation due to exogenous factors (such as aging, environmental factors, and oxidative stress) is closely related to the progression of PD [4, 5]. Neuroinflammation that activates the microglia and astrocytes and facilitates subsequent infiltration of the blood-brain barrier (BBB) can cause neurovascular dysfunction, which in turn plays not only a proinflammatory but also proangiogenic role [6]. Neurovascular changes interact in an important way with the neurodegenerative process in idiopathic PD [7], vascular parkinsonism [8], Alzheimer's disease (AD), and dementia [6] and lead to structural brain atrophic changes [9, 10]. In this study, we sought to clarify the association between vascular inflammatory markers and specific levels of brain atrophic changes in order to clarify the pathogenesis of PD.

Abnormal neurovascular alterations involve BBB dysfunction, resulting in neoangiogenesis and even hemorrhage [11]. Angiogenesis may compromise the function of the BBB, which could contribute to ongoing neuroinflammation. This may result in turn in dopaminergic neuron loss due to neutrophil infiltration, leading to neuronal damage in the substantia nigra and increased BBB permeability, resulting in a vicious cycle [12, 13]. Moreover, microglial cells release proinflammatory cytokines and act on the endothelium of BBB cells to stimulate the upregulation of vascular cell adhesion molecule-1 (VCAM-1) and intercellular adhesion molecule-1 (ICAM-1) [14] and play a role in the transmigration of leukocytes and related subtypes to the central nervous system (CNS). The elevation of cell adhesion molecules is an indicator of BBB integrity [15] and is related to disease severity in PD [16, 17] and white matter disintegrity in AD dementia [18]. With regard to angiogenesis, microRNAs (miRNAs) are highly conserved, single-stranded, noncoding small RNAs that regulate gene expression at the posttranscriptional level. A previous study revealed that miRNA-29a modulates the angiogenic properties of human endothelial cells both in vitro and in vivo [19], while another study showed that miRNA-29a is downregulated in naïve PD patients but overexpressed in L-dopa-treated PD patients [20]. Such serum vascular markers can be further evaluated and have a potential value as biomarkers in the evaluation of BBB dysfunction and the angiogenesis-associated disease progression of PD.

Neurovascular status changes in the brain due to BBB dysfunction and aberrant angiogenesis cause white matter lesions (leukoaraiosis) [7], vessel regression, and brain hypoperfusion [21]. Increasingly severe small vessel disease is associated with increases in both white matter hyperintensities and degrees of brain atrophy [9], while both small vessel disease and gray matter atrophy are correlated with cognitive impairment in PD and Parkinsonian signs. In this study, we used structural MRI to measure brain gray matter volumes and provide imaging evidence of neuron loss in PD. Volumetric MRI measures reflect the accumulated effects of different pathological factors in PD, which may account for why such measures are good predictors of disease progression.

PD is diagnosed anatomopathologically, and a definite diagnosis can only be reached by post-mortem finding. This study hypothesized that vascular inflammation, as determined by vascular adhesion molecule- and angiogenesis-related miRNA analysis, may reflect neuroinflammatory changes in PD that ultimately result in specific brain gray matter changes and are correlated with clinical disease severity. After statistical analysis, the results link brain gray matter volumes with the levels of selected peripheral vascular inflammatory biomarkers and disease severity.

## 2. Materials and Methods

### 2.1. Participants

The protocol of this study conformed to the ethical guidelines of the 1975 Declaration of Helsinki reflected in a priori approval by the Chang Gung Memorial Hospital human research committee. Thirty-three patients (11 males and 22 females; mean age 63.1 ± 9.09 years) with idiopathic PD diagnosed according to the United Kingdom Brain Bank criteria [22] by an experienced neurology specialist and without a history of other neurologic or psychiatric illness or psychotropic medication use were prospectively enrolled through the Neurology Department of Chang Gung Memorial Hospital. For each patient, the time of diagnosis and the duration of the disease were recorded. The disease onset was defined as the time of the first recalled motor symptoms, such as tremor, bradykinesia, and rigidity, in the pretreatment phase of the disease.

The assessments were performed at least 12 h after the last dose of dopaminergic medication (off state). Each patient's disease severity and functional status were evaluated using the Mini-Mental State Examination (MMSE), which was used to assess the general cognitive functions of the subjects [23]; the Unified Parkinson's Disease Rating Scale (UPDRS); the modified Hoehn and Yahr Staging Scale (HY-stage); and the Schwab and England Activities of Daily Living Scale (SE-ADL). The UPDRS is the scale most commonly used to follow the longitudinal course of PD, with the UPDRS score being determined by interview and clinical observation [24]. The modified HY-stage is a 5-point ordinal scale that provides a global assessment of PD severity based on clinical findings and functional disability [25] and is commonly used to describe the progression of PD from HY-stage 1 through stage 5. The SE-ADL estimates the abilities of PD patients relative to completely independent and healthy individuals; a score of 100% indicates a completely independent patient, while a score of 0% indicates a bedridden individual with only vegetative functions.

For comparison, 27 sex- and age-matched healthy subjects (7 males and 20 females; mean age 59.2 ± 9.71 years) without a medical history of neurologic disease or psychiatric illness, alcohol or substance abuse, or head injury and with similar levels of education were recruited from the hospital as a control group. The hospital's Institutional Review Committee on Human Research approved the study protocol, and all of the participants or their guardians provided written informed consent. In addition, previous demographic data demonstrate that males have a two-fold higher incidence of PD diagnosis compared to females [26]. In clinical presentation, males have more rigidity and rapid eye movement behavior disorder, whereas more females than males exhibit dyskinesia and depression. In single-photon emission computed tomography (SPECT) imaging, females had higher levels of striatal uptake binding than males at the time of symptom onset and a more benign preclinical phase of PD, possibly due to protective effects exerted by estrogen [27, 28]. In this study, no gender significance between the PD patient group and normal control group was evident, while statistical correlation analysis was adjusted for the effects of age and sex differences.

### 2.2. MRI Acquisition

Volumetric structural MRI scans were acquired on a GE Signa 3 T whole-body MRI scanner (General Electric Healthcare, Milwaukee, WI, USA) using an eight-channel phase array head coil at Kaohsiung Chang Gung Memorial Hospital in Taiwan. Whole-brain three-dimensional T1-weighted images were collected for each participant using an inversion-recovery fluid-attenuated fast spoiled gradient-recalled echo pulse sequence with the following imaging parameters: repetition time (TR)/echo time (TE)/inversion time (TI) =9.5/3.9/450 ms, flip angle = 15 degrees, number of excitations (NEX) = 1, field of view (FOV) = 240 × 240 mm^2^, matrix size = 512 × 512, voxel size = 0.47 × 0.47 × 1.3 mm^3^, and slice number = 110 axial slices (without interslice gaps). In order to identify any brain abnormalities, an additional volumetric axial T2-weighted fast spin-echo sequence (TR/TE = 4200/102 ms, echo train length = 18, NEX = 2, FOV = 240 mm^2^, slice thickness = 5 mm, matrix size = 320 × 256, and 18 slices) and axial T2-weighted inversion-recovery fluid-attenuated sequence (TR/TE/TI = 8000/100/2000 ms, NEX = 1, FOV = 240 mm^2^, slice thickness = 5 mm, matrix size = 320 × 256, and 18 slices) were used in the same imaging session.

### 2.3. VBM Analyses

VBM analyses were conducted using the VBM12 toolbox (Christian Gaser, University of Jena; http://dbm.neuro.uni-jena.de/vbm12/) in SPM12 (Wellcome Department of Cognitive Neurology, London, UK) running in MATLAB R2012a (MathWorks, Natick, MA). The T1-weighted images were segmented into gray matter, white matter, and cerebrospinal fluid (CSF) images. The total intracranial volumes (TIVs) were obtained from the native space images. The images were normalized to Montreal National Institute (MNI) space using the high-dimensional DARTEL normalization procedure [29]. The images were modulated using the Jacobian determinants derived from the normalization procedure. A 10 mm full width at half maximum (FWHM) Gaussian kernel was used to smooth the images to improve the signal-to-noise ratio.

The images were analyzed with SPM12 using the general linear model (GLM). Statistical analysis was performed among the PD and control groups, with age, sex, and total intracranial volume (TIV) used as covariates, using a two-sample *t*-test in SPM12. Results were considered significant under the criteria of family-wise error- (FWE-) corrected *P* value < 0.05 based on the results of the Monte Carlo simulation (3dClusterSim with the following parameters: single voxel *P* value < 0.005, FWHM = 8 mm with a GM mask, and 10,000 simulations). Visualization of the results was performed using the xjView toolbox (http://www.alivelearn.net/xjview).

### 2.4. Laboratory Measurements of Vascular Inflammatory Markers in the Peripheral Circulation

#### 2.4.1. Blood Sampling

For each participant, blood was drawn by venipuncture from the forearm on the same day as the MRI study and neuropsychological testing.

#### 2.4.2. Measurements of Adhesion Molecule Levels

Blood samples were collected by venipuncture into Vacutainer SST tubes (BD, Franklin Lakes, NJ, USA). Blood was allowed to clot at room temperature for a minimum of 30 minutes. The clots were then removed by centrifugation at 3000 rpm for 10 minutes at 4°C. All serum samples were collected after centrifugation, isolated, and immediately stored at −80°C in multiple aliquots. The quantization of markers of the endothelium, soluble ICAM-1 and VCAM-1 (R&D Systems), in the serum was measured by an enzyme immunoassay. In the assay, standards, controls, and unknown samples were incubated in microtitration wells that were coated with antibodies of the markers (anti-ICAM-1 and anti-VCAM-1). After incubation and washing, the wells were treated with another anti-Ag detection antibody labeled with the enzyme horseradish peroxidase (HRP). After a second incubation and washing step, the wells were incubated with the substrate tetramethylbenzidine (TMB). An acidic stopping solution was then added, and the degree of enzymatic turnover of the substrate was determined by dual-wavelength absorbance measurement at 450 and 620 nm. The absorbance was directly proportional to the concentration of antigens present. A set of antigen standards was used to plot a standard curve of absorbance versus antigen concentrations, from which the antigen concentrations in the unknowns could be calculated.

#### 2.4.3. miRNA Analysis Methods

We analyzed the expressions of miRNA-29a-3p and miRNA-22-5p and the candidate normalizer miRNA-451. Blood samples were obtained with EDTA-containing tubes. Total RNA from 300 *μ*L plasma was isolated using the mirVana PARIS Kit (Ambion, Austin, TX, USA) according to the manufacturer's instructions. Blood samples were centrifuged at 3000 rpm for 10 min to pellet cellular debris. We used the supernatant for RNA extraction, after the addition of denaturing solution (Ambion, Austin, TX, USA) and after 4 *μ*L RNA isolation spike-in mix (UniSp2, UniSp4, and UniSp5) (miRCURY LNA™ Universal RT miRNA PCR, RNA Spike-In Kit, Exiqon) was spiked into each sample for normalization of the variation between the samples. In brief, 2 *μ*L total RNA was collected and pooled from the samples from the same fluid type, and the cDNAs were produced using the Universal cDNA Synthesis Kit (EXIQON). After forty rounds of diluting the amounts of the PCR product, the amplification was determined by the level of fluorescence emitted by SYBR Green (ExiLENT SYBR Green Master Mix, EXIQON). The endogenous abundance of miRNAs was detected on the Roche LightCycler® 480 real-time PCR platform (Roche Diagnostics Ltd., Mannheim, Germany). The relative expression level of each miRNA was expressed using the 2^−*ΔΔ*Ct^ method, where ΔCt = Ct_miRNA_ − Ct_miR451a_.

### 2.5. Statistical Analysis

The demographic data, including age and sex, were compared among the study groups by applying the 2-sample Student *t*-test or the Mann-Whitney test, where appropriate, and are reported as mean ± the standard deviation (SD). The significance of differences in miRNAs, vascular adhesion molecules, and disease severity was analyzed by analysis of covariance (ANCOVA) with the participants' age and sex as covariates. The associations between the vascular markers and variables were tested by partial correlation analysis after adjustments for age and sex. The threshold for all statistical significance was set at *P* < 0.05. SPSS software (SPSS V.17, Chicago, IL, USA) was used to perform all the statistical analyses.

## 3. Results

### 3.1. Baseline Characteristics of the Study Patients

The baseline characteristics of the two groups (Table 1) showed no significant differences in terms of age or sex. The clinical disease severity was measured using the UPDRS (UPDRS I, UPDRS II, UPDRS III, and UPDRS 176), modified HY-stage, SE-ADL, and MMSE. Their MMSE scores were calculated and showed a significant difference between the two groups. The other clinical disease severity evaluations focused on the patient group, and so comparisons with the normal healthy group could not be performed.

### 3.2. Vascular and Adhesion Molecule and miRNA Profiles in the PD and Control Groups

The laboratory data, presented as a mean value with standard deviation for both groups, showed that the VCAM-1 expression was significantly higher in the PD patients than in the controls (820.57 ± 316.8 ng/mL vs. 663.21 ± 137.6 ng/mL). The levels of circulating soluble VCAM-1 and ICAM-1 in control subjects are within normal limit. In contrast, the expression of ICAM-1 did not significantly differ between the two groups. These results also demonstrate that miRNAs can be detected in total peripheral blood and differentially expressed between normal and PD subjects. MicroRNAs (including miR-22 and miR-29a) were significantly upregulated in the PD group compared with the normal group (*P* = 0.003 and *P* = 0.004, respectively) (Table 2).

### 3.3. Gray Matter Volume Reduction in Patients with PD

**Figure 1:**
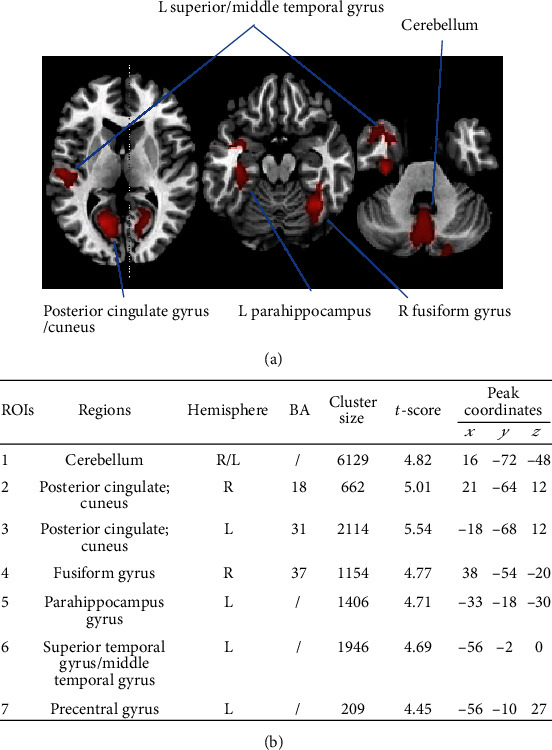
Anatomical locations with significant differences in gray matter density. (a) Significant (cluster level statistics, *P* value < 0.05, FWE-corrected) regional gray matter volume reduction in a patient with Parkinson's disease was revealed by whole-brain VBM analysis. (b) The coordinates *x*, *y*, and *z* refer to the anatomical location, indicating the standard stereotactic space as defined by Montreal Neurological Institute. The *t*-score of the voxel with the strongest group effect in a given cluster is also listed. Abbreviations: ROIs: regions of interest; BA: Brodmann area; L: left; R: right; FWE: family-wise error; MNI: Montreal Neurological Institute; VBM: voxel-based morphometry.

Compared to the healthy controls, the PD patients showed a significant gray matter volume reduction in the following brain regions: (ROI 1) the cerebellum, (ROI 2 and 3) bilateral posterior cingulate gyrus, (ROI 3) left limbic lobe (hippocampus and uncus), (ROI 4) right fusiform gyrus, (ROI 5) left parahippocampus, (ROI 6) left superior and middle temporal gyrus, and (ROI 7) left precentral gyrus. The sum of all the above brain gray matter volume changes was calculated as the whole-brain gray matter volume change (ROI 8). We did not find increased gray matter volume in the PD patients compared with the healthy controls (Figure 1).

### 3.4. Correlations of Gray Matter Atrophy, miRNAs, Cell Adhesion Molecule, and Disease Severity Levels

The VCAM-1 expression was negatively correlated with the gray matter volume of the left parahippocampus (*r* = −0.290, *P* = 0.043), left superior/middle temporal gyrus (*r* = −0.291, *P* = 0.042), and whole-brain ROI cluster (*r* = −0.314, *P* = 0.028) (Table 3). Moreover, the VCAM-1 expression was positively correlated with the clinical disease severity score (Table 4). The miRNA-29a expression was negatively correlated with the gray matter volume of the cerebellum (*r* = −0.331, *P* = 0.020), right fusiform gyrus (*r* = −0.353, *P* = 0.007), and whole-brain ROI cluster (*r* = −0.337, *P* = 0.010) (Table 3).

The gray matter volume of the right posterior cingulate/cuneus region (ROI 2) was negatively correlated with the UPDRS II, UPDRS III, and UPDRS 176 results (*r*, *P* = −0.385, 0.032; -0.388, 0.031; and -0.382, 0.034, respectively), and that of the left posterior cingulate/cuneus region (ROI 3) was negatively correlated with the UPDRS III and UPDRS 176 results (*r*, *P* = −0.041, 0.025 and -0.387, 0.031, respectively). The gray matter volume of the left parahippocampus gyrus region (ROI 5) was also negatively correlated with the UPDRS II, UPDRS III, and UPDRS 176 results (*r*, *P* = −0.423, 0.018; -0.389, 0.031; and -0.410, 0.022, respectively). The gray matter volume of the left precentral gyrus region (ROI 7) was likewise negatively correlated with the UPDRS II, UPDRS III, and UPDRS 176 results (*r*, *P* = −0.446, 0.012; -0.370, 0.040; and -0.387, 0.031, respectively).

## 4. Discussion

Consistent with our vascular hypothesis, the patients with PD experienced higher serum vascular adhesion molecule levels, as well as high expression levels of angiogenic miRNAs, suggesting the occurrence of vascular inflammation and related neuroinflammation. Furthermore, we identified gray matter atrophy throughout much of the cortex, including in anatomical locations that typically appear to be particularly sensitive to the effects of inflammation, such as the cerebellum, bilateral posterior cingulate gyrus, left parahippocampus, and left temporal gyrus [30]. We further demonstrated, for the first time, that gray matter volume atrophy in the parahippocampus correlated with VCAM-1 levels and disease severity scores. As for disease progression, VCAM-1 levels were also found to be positively correlated with disease severity. The correlations of the vascular inflammatory parameters with brain gray matter atrophy in the dominant side of the parahippocampus and temporal lobe may mirror the relationship between vascular inflammatory factors and neuroinflammation-related brain atrophy during the neurodegenerative process in PD.

### 4.1. The Changes of Brain Gray Matter Atrophy Reflect the Neuronal Loss That Occurs during Disease Progression

Volumetric MRI measures of whole-brain and regional atrophy provide indirect assessments of neuronal loss, and correlations of such measures with clinical disease progression have been proposed as a useful radiological tool of neurodegeneration in PD [31–33]. Data from our previous MRI studies suggest a spatial pattern of regional volume loss in PD that closely parallels the respiratory dysfunction, perceptual impairment, and systemic oxidative stress seen in PD [10, 34]. The PD signature of regional atrophy observed in this study was characterized by predominant involvement of the left parahippocampus as well as the bilateral posterior cingulate gyrus, fusiform, left temporal gyrus, and cerebellum, with gray matter volume changes in these regions also being correlated with disease severity levels (as measured by UPDRS II, III, and 176 scores). These atrophic areas include key components of mental processing (the superior temporal gyrus) [35], memory formation (the hippocampus), high-level visual processing (the fusiform cortex) [36], and social cognition and affective processing (the cerebellum) [37]. A previous study revealed that poor performance on visuoperceptual tests was significantly associated with gray matter decreases in the fusiform, the parahippocampus, and the middle occipital gyrus [38], while another study reported that decreased cortical thickness was also related to cognitive deterioration (including in the storage of prior experiences, integration of external perceptions, and semantic processing) [39]. Recent meta-analysis comparing PD patients without cognitive impairment (PD-NCI), PD patients with mild cognitive impairment (PD-MCI), and PD patients with dementia (PDD) [40] revealed that PD-MCI have left temporal lobe atrophy compared with PD-NCI. The left temporal lobe and left parahippocampal gyrus showed significant atrophy in the PDD group compared with PD-ND (nondementia), results which support our findings. Gray matter atrophy has been widely studied in previous PD research studies, but what factors cause brain cortex atrophy? Recent research has revealed that the progression of cognitive impairment in PD patients occurs through two steps: hypometabolism due to cortical blood flow perfusion deficits [41, 42] and corticolimbic gray matter loss [43]. The hypometabolism may be due to lower cerebral profusion and related to vascular factors. Lower perfusion in PD and PD dementia patients has been demonstrated by using arterial spin labeling (ASL) MRI [42]. In the brain parenchyma, neurovascular changes may interact with the neurodegenerative process in idiopathic PD, with markers of neurovascular status including white matter changes and cerebral blood flow [7]. Gray matter volume atrophy is closely associated with underlying white matter changes and may be correlated with vascular factors like neoangiogenesis and BBB dysfunction [44, 45].

Our previous study reported that PD patients exhibited widespread striatocortical functional network alterations to the left hippocampus and right cerebellum, associated with a decline in the dopamine transporter ratio of the striatum, as detected by 99mTc-TRODAT-1 SPECT/CT imaging [46]. These results support our findings and demonstrate that PD influences not only the nigrostriatal system but also the mesolimbic system. The dopaminergic system consists of two distinct nominal systems: a nigrostriatal system for motor function and a mesolimbic system for motivation and reward functions. While the two pathways seem to have independent anatomical origins, a previous study suggests that dopamine in both nigrostriatal and mesocorticolimbic terminal fields participates in the defining property of rewarding events and the reinforcement of memory consolidation [47] and plays roles in axonal damage and loss of connectivity in early Parkinson's disease [48]. Another study focused on corticolimbic gray matter loss revealed the combined effect of the two dopaminergic pathways in PD patients with mild cognitive impairment [43]. Our study results are compatible with corticolimbic gray matter atrophy in the parahippocampus, cerebellum, and occipital lobe, indicating neuron loss related to dopaminergic system dysfunction in PD patients.

### 4.2. VCAM-1 and miRNA Profiles as Vascular Inflammation Indicators in PD Patients

**Figure 2:**
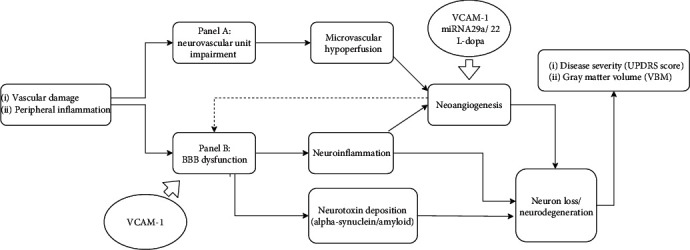
Vascular factors of Parkinson disease, modified by two hit vascular model references [6]. Peripheral inflammation with vascular factor-like hypertension, diabetes, aging process, and genetic factors for PD, leading to neurovascular unit (NUV) impairment (panel A) and BBB dysfunction (panel B). In panel A, abnormal neurovascular alterations cause changes in vascular perfusion in the cerebral blood flow and oxygen consumption and cerebral flow hypoperfusion with small vessel atherosclerosis, further leading to neoangiogenesis and neuroinflammation. In panel B, peripheral inflammation and the aging process can cause BBB dysfunction, with inflammatory factors infiltrating into the neurons and adjacent supporting tissue. Inflammation also causes neurotoxin accumulation in brain tissues and vascular damage that changes microvessel integrity, leading to microvascular hypoperfusion (oligaemia) and inducing neoangiogenesis, which in turn result in macrobrain structural changes like white matter hypertrophy and gray mater atrophy, which are in turn correlated with clinical disease progression. Abbreviations: VCAM-1: vascular cell adhesion molecule-1; UPDRS: Unified Parkinson's Disease Rating Scale; VBM: voxel-based morphology.

According to Figure 2, disease progression and brain gray matter volume atrophy are the combined end results of peripheral inflammation and BBB dysfunction and their subsequent sequelae. Peripheral inflammation and the aging process can cause BBB dysfunction and cause inflammatory factors to infiltrate into neurons and adjacent supporting tissue. Inflammation also causes vascular damage that changes microvessel integrity, resulting in microvascular hypoperfusion and inducing neoangiogenesis, which in turn result in macrobrain structural changes like WM hypertrophy and, finally, neurodegeneration and clinical disease progression [6, 49].

In this study, the significantly higher levels of VCAM-1 in the PD patients were important clues suggesting that underlying vascular factors contribute to neuroinflammation. In brain tissues, dysfunction of the BBB accompanied by microglial activation and neutrophil infiltration leads to the loss of dopaminergic neurons caused by programmed cell death [13, 50, 51]. Under conditions of inflammation, the microglial release of proinflammatory cytokines, including tumor necrosis factor-alpha (TNF-*α*), interleukin- (IL-) 1*β*, and transforming growth factor-*β*, acts on the endothelium of BBB cells to stimulate the upregulation of VCAM-1 and ICAM-1, causing the transmigration of leukocytes and related subtypes from the blood vessels into the CNS, promoting neuroinflammation [52, 53]. Cell-bound VCAM-1 allows the human brain microvascular endothelium to control immune cell trafficking across the BBB. It is upregulated in the inflammatory-active brain lesions of patients with multiple sclerosis (MS) [54], neurodegenerative diseases like AD [18], and atherosclerosis [55]. Not only is it correlated with the proinflammatory cytokines involved in BBB dysfunction, but also according to one in vitro study, soluble VCAM-1 directly impairs the integrity of human brain endothelial barriers, causing increased permeability that compromises the barrier function by inducing intracellular signaling by integrin alpha-4 [15]. Meanwhile, in normal elderly healthy individuals, elevated plasma VCAM-1 is associated with impaired cerebrovascular function and mobility impairments [56]. An association between VCAM-1 and CO_2_-related cerebral vasoreactivity, as well as gray matter atrophy, has also been reported in diabetic subjects [57], while elevated VCAM-1 is also associated with rather large effects on white matter hyperintensities [58]. In this study, the levels of soluble VCAM-1 were positively correlated with the clinical PD disease severity scores. Therefore, VCAM-1 may serve an important role in BBB dysfunction and as a clinical biomarker of PD and its clinical consequences.

BBB dysfunction may also be associated with angiogenesis [59]. In the brain, immature vessels likely lack the full characteristics of the BBB, including the development of tight junctions, the recruitment of pericytes, and the formation of a glial limitans. Thus, angiogenesis may compromise the function of the BBB, which could contribute in turn to ongoing neuroinflammation by allowing peripheral molecules and immune cells to access the brain parenchyma. Indeed, increased CSF levels of vascular endothelial growth factor (VEGF) have been seen in PD patients and associated with BBB permeability and white matter lesions and further related to clinical symptoms such as gait difficulties and orthostatic hypotension, indicating increased BBB permeability and angiogenesis [60].

In this study, the angiogenesis-related miRNA-29a and miRNA-22 were upregulated in the PD patients, suggesting an underlying and ongoing angiogenesis process. miRNA-22 regulates inflammation and angiogenesis by targeting VE-cadherin [61], and miRNA-29a can modulate the angiogenic properties of human endothelial cells [19] and be upregulated by proinflammatory cytokine transforming growth factor-*β* (TGF-*β*) [62]. The miRNA is also involved in neuron growth [63] and atherosclerosis formation [64]. An *in vitro* study found that miRNA-29a overexpression promoted the formation of new blood vessels, while miR-29a suppression completely blocked TGF-*β*1-stimulated angiogenesis [62]. TGF-*β*1 expression was increased in striatal neurons and in activated microglia on the lesion side of a 6-hydroxydopamine-induced PD mouse model in one *in vivo* study [65] and was elevated in the CSF fluid of PD patients in an *in vitro* study, indicating that it acts as a neuroprotective factor in injured brains. Another previous study revealed the downregulation of miRNA-29a in treatment-naïve PD patients [66], but upregulation in L-dopa-treated PD patients has also been observed [20]. In another neurodegenerative disease, AD, miRNA-29a was found to be upregulated in blood-CSF samples [67] but decreased in brain tissue [68]. In this study, the upregulation of miR-29a may have been due to the combination of hypoxia-related neoangiogenesis mechanisms and L-dopa treatment-related angiogenesis [69]. Our previous study revealed that the hypoxia-like status in obstructive sleep apnea may cause alterations to regional cerebral blood flow [70]. Hypoxia-ischemia can induce profound nonpreferential and immediate mobilization of leucocytes into the circulation, along with a significant influx of neutrophils into the brain with subsequent neuroinflammation [71]. Another factor of hypoxia in the microstructures of the brain is the change in neurovascular unit (NVU) integrity caused by L-dopa treatment that in turn causes regional cerebral metabolism and blood flow changes, which are also related to the angiogenesis and increased BBB permeability induced by VEGF regulation, and is reflected in the clinical result of L-dopa-induced dyskinesia. Our previous study using MRI arterial spin labeling also showed vascular changes in PD patients receiving acute L-dopa treatment. Hypoperfusion in the occipital, parietal, and cerebellar regions, as well as extensive neocortical hypoperfusion, in PD dementia patients has also been noted. In this study, the upregulated expression of miRNA-29a was positively correlated with cerebellar atrophy and whole-brain gray matter atrophy. As for disease progression under L-dopa treatment, evidence suggests that miRNA-29a expression level changes play a role in the microvascular remodeling process, brain morphology, and vascular flow changes.

### 4.3. Partial Correlations among Disease Severity, Specific Brain Atrophy, and Vascular Markers

The PD signature of regional atrophy in this study was characterized by the predominant involvement of the left parahippocampus, bilateral posterior cingulate gyrus, fusiform, left temporal gyrus, and cerebellum, in addition to being correlated with disease severity, findings which were also supported by our previous studies [72, 73]. After controlling for age and sex, volume changes in the left parahippocampus were weakly to moderately correlated with VCAM-1 expression levels. The parahippocampus is a key component of the human ventral temporal cortex, which has the function of visual categorization and recognition [74], is part of the salience network that functions in memory retrieval [75], and is part of the default mode network involved in memory encoding [76]. Our previous study demonstrated that mesial temporal network degeneration interacts with systemic oxidative stress and cognitive impairment in PD patients [73]. In parahippocampal gyrus brain tissue, the accumulation of alpha-synuclein is directly related to the level of beta-amyloid and the Braak tangle stage and can predict cognitive status in PD dementia patients [77]. Capillary vascular morphology and degeneration have been identified in human brain tissue from PD patients through immunohistochemical staining [78] and related to changes in the expression of hypoxia- and angiogenesis-related genes [79]. Vascular degeneration in PD appears to be the result of endothelial cell degeneration, even as the capillary basement membrane is retained. Increased string vessel formation suggests a role for vascular hypoperfusion in the progression of PD, while leakage of the BBB may be associated with astrocytosis due to inflammatory stimulation, which appears to be a pathological change secondary to the initial lesion in PD [78]. BBB permeability change may cause brain microvascular change, affecting interstitial flow, waste debris clearance, and molecule diffusion/transport, further reducing the drug delivery effect [80]. As shown in Table 4 of this study, VCAM-1 expression was moderately correlated with all the disease severity scores and weakly to moderately correlated with whole-brain atrophy and left parahippocampal gyrus atrophy, supporting the role of VCAM-1 as a vascular marker in PD and providing further evidence of the interactions among neuroinflammation, neurovascular unit integrity, BBB dysfunction, and subsequent PD disease progression. For this reason, the combination of vascular markers with brain gray matter atrophy can integrate more components of PD pathogenesis, suggesting a potential prognostic marker to monitor PD progression and therapeutic effectiveness.

### 4.4. Limitations

The interpretation of the findings presented here must be tempered by some of the limitations of the present study. First, the patients who participated were recruited from a single tertiary center and so may not be representative of all PD populations. Second, this study was a cross-sectional and small-sized study, while the sample power of this study is underpowered (49% power); thus, the significance of the results should be interpreted cautiously [81]. Further causal relationships among grey matter volume changes, clinical disease severity, and vascular inflammatory markers may need to be delineated by future longitudinal studies. Third, the measurement of only a few vascular inflammatory biomarkers cannot be considered a valid tool for exploring the multifaceted, complex BBB dysfunction and angiogenesis imbalance in PD. The microenvironment interactions in the BBB region and brain parenchyma due to CNS and systemic oxidative stress can be affected by individual genetic variations and physical exercise, and these interactions were not well evaluated in the present study. In addition, recent research suggests that network-based rather than regional anatomic involvements contribute to the neurodegeneration and disease severity seen in PD, such that assessing structural or functional connectivity between brain regions associated with neurovascular changes would clarify the complex neural network involved in vascular factor and BBB dysfunction.

## 5. Conclusions

Although extensive structural alterations might occur in PD, our results highlight the possibility that changes in only some vulnerable anatomies, such as the temporal lobe and parahippocampus, might be correlated with the vascular inflammation seen in certain phenotypes of PD. This model has important implications for understanding and providing more evidence that vascular inflammation may result in the development of PD and might provide novel targets for candidate neuroprotective therapies.

## Figures and Tables

**Table 1:** Demographic data of PD patients and controls.

Parameters	PD patients (*n* = 33)	Controls (*n* = 27)	*P*
Age (year) (mean ± SD)	63.1 ± 9.09	59.2 ± 9.71	*0.072*
Sex (M, F)	11, 22	7, 20	*0.160*
UPDRS I	3.1 ± 3.44		
UPDRS II	9.5 ± 6.45		
UPDRS III	24.7 ± 16.59		
UPDRS 176	37.3 ± 25.34		
Modified HY-stage (maximum stage is 5)	1.70 ± 0.89		
SE-ADL (minimum score 0 suggests the presence of only vegetative function)	83.94 ± 14.78		
MMSE	23.76 ± 4.81	28.07 ± 1.82	*0.010* ^∗^

Abbreviations: PD: Parkinson's disease; UPDRS: Unified Parkinson's Disease Rating Scale; modified HY-stage: modified Hoehn and Yahr Staging Scale; SE-ADL: Schwab and England Activities of Daily Living Scale; MMSE: Mini-Mental State Examination. Data are presented as mean ± SD. ∗ indicates a *P* value of less than 0.05.

**Table 2:** Cell adhesion molecule and microRNA profile level of PD patients and controls.

Parameters	PD patients (*n* = 33)	Controls (*n* = 27)	*P*
Cell adhesion molecule parameters			
VCAM-1 (ng/mL)	820.57 ± 316.8	663.21 ± 137.6	*0.025* ^∗^
ICAM-1 (ng/mL)	200.16 ± 77.56	202.79 ± 68.74	*0.898*
miRNA fold change (FC)	PD vs. control		
miRNA-22	1.99		*0.004* ^∗^
miRNA-29a	1.70		*0.003* ^∗^

VCAM-1: vascular cell adhesion molecule-1; ICAM-1: intracellular cell adhesion molecule-1; miRNA: microribonucleic acid. Data are presented as mean ± SD. ∗ indicates a *P* value of less than 0.05.

**Table 3:** Correlation analysis of levels of vascular cell adhesion molecule, miRNAs, and disease severity to gray matter atrophy in the PD group after controlling for age and sex.

PD brain region																
	Cerebellum		Posterior cingulate/cuneus		Posterior cingulate/cuneus		Fusiform gyrus		Parahippocampus gyrus		Superior temporal gyrus/middle temporal gyrus		Precentral gyrus		Whole brain	
ROI	1		2		3		4		5		6		7		All clusters	
Hemisphere			R		L		R		L		L		L			
	*r*	*P*	*r*	*P*	*r*	*P*	*r*	*P*	*r*	*P*	*r*	*P*	*r*	*P*	*r*	*P*
Cell adhesion marker																
VCAM-1 (ng/ml)									-0.290	*0.043* ^∗^	-0.291	*0.042* ^∗^			-0.314	*0.028* ^∗^
MicroRNA																
miRNA-29a	-3.300		0.022^∗^				-0.353	*0.007* ^∗^							-0.337	*0.010* ^∗^
Disease severity																
UPDRS I																
UPDRS II			-0.385	*0.032* ^∗^					-0.423	*0.018* ^∗^			-0.446	*0.012* ^∗^		
UPDRS III			-0.388	*0.031* ^∗^	-0.401	*0.025* ^∗^			-0.389	*0.031* ^∗^			-0.370	*0.040* ^∗^		
UPDRS 176			-0.382	*0.034* ^∗^	-0.387	*0.031* ^∗^			-0.410	*0.022* ^∗^			-0.387	*0.031* ^∗^		

The results of partial correlation analysis were illustrated as correlation coefficients and corresponding *P* values (controlling for age and sex, Bonferroni corrected ^∗^*P* < 0.05). Abbreviations: PD: Parkinson's disease; VCAM-1: vascular cell adhesion molecule-1; UPDRS: Unified Parkinson's Disease Rating Scale.

**Table 4:** Associations between vascular cell adhesion molecule-1 (VCAM-1) levels and disease severity scores after controlling for age and sex.

Disease severity score	Correlation coefficient	*P* value
UPDRS I	0.461	0.005
UPDRS II	0.567	0.027
UPDRS III	0.535	0.008
UPDRS 176	0.563	0.005
HY-stage	0.418	0.047
SE-ADL	-0.604	0.002

The results of partial correlation analysis were illustrated as correlation coefficients and corresponding *P* values (controlling for age and sex, Bonferroni corrected *P* < 0.05). Abbreviations: PD: Parkinson's disease; UPDRS: Unified Parkinson's Disease Rating Scale; modified HY-stage: modified Hoehn and Yahr Staging Scale; SE-ADL: Schwab and England Activities of Daily Living Scale.

## Data Availability

The datasets generated and analyzed during the current study are not publicly available due to the restriction from the ethics review board but are available from the corresponding author on reasonable request.

## Abbreviations

ANCOVA: Analysis of covariance
BBB: Blood-brain barrier
GMV: Gray matter volume
miRNAs: MicroRNAs
MMSE: Mini-Mental State Examination
NC: Normal control
PD: Parkinson's disease
SPM: Statistical Parametric Mapping software program
SPSS: Statistical Package for the Social Sciences
TIV: Total intracranial volume
UPDRS: Unified Parkinson's Disease Rating Scale
VBM: Voxel-based morphometry
VCAM-1: Vascular cell adhesion molecule-1.

## References

[1] Habibi E., Masoudi-Nejad A., Abdolmaleky H. M., Haggarty S. J. (2011). Emerging roles of epigenetic mechanisms in Parkinson's disease. *Functional & Integrative Genomics*.

[2] Braak H., Tredici K. D., Rüb U., de Vos R. A. I., Jansen Steur E. N. H., Braak E. (2003). Staging of brain pathology related to sporadic Parkinson's disease. *Neurobiology of Aging*.

[3] Shults C. W. (2006). Lewy bodies. *Proceedings of the National Academy of Sciences of the United States of America*.

[4] Dias V., Junn J., Mouradian M. M. (2013). The role of oxidative stress in Parkinson’s disease. *Journal of Parkinson’s Disease*.

[5] Ali S. F., Binienda Z. K., Imam S. Z. (2011). Molecular aspects of dopaminergic neurodegeneration: gene-environment interaction in parkin dysfunction. *International Journal of Environmental Research and Public Health*.

[6] Nelson A. R., Sweeney M. D., Sagare A. P., Zlokovic B. V. (2016). Neurovascular dysfunction and neurodegeneration in dementia and Alzheimer's disease. *Biophysica Acta (BBA) - Molecular Basis of Disease*.

[7] Al-Bachari S., Vidyasagar R., Emsley H. C. A., Parkes L. M. (2017). Structural and physiological neurovascular changes in idiopathic Parkinson's disease and its clinical phenotypes. *Journal of Cerebral Blood Flow and Metabolism*.

[8] Korczyn A. D. (2015). Vascular parkinsonism--characteristics, pathogenesis and treatment. *Nature Reviews. Neurology*.

[9] Lambert C., Benjamin P., Zeestraten E., Lawrence A. J., Barrick T. R., Markus H. S. (2016). Longitudinal patterns of leukoaraiosis and brain atrophy in symptomatic small vessel disease. *Brain*.

[10] Lin W.-C., Chou K.-H., Lee P.-L. (2015). Brain mediators of systemic oxidative stress on perceptual impairments in Parkinson's disease. *Journal of Translational Medicine*.

[11] Chen C.-C. V., Chen Y.-C., Hsiao H.-Y., Chang C., Chern Y. (2013). Neurovascular abnormalities in brain disorders: highlights with angiogenesis and magnetic resonance imaging studies. *Journal of Biomedical Science*.

[12] Rossi B., Angiari S., Zenaro E., Budui S. L., Constantin G. (2011). Vascular inflammation in central nervous system diseases: adhesion receptors controlling leukocyte-endothelial interactions. *Journal of Leukocyte Biology*.

[13] Ji K. A., Eu M. Y., Kang S. H., Gwag B. J., Jou I., Joe E. H. (2008). Differential neutrophil infiltration contributes to regional differences in brain inflammation in the substantia nigra pars compacta and cortex. *Glia*.

[14] Whitton P. S. (2007). Inflammation as a causative factor in the aetiology of Parkinson's disease. *British Journal of Pharmacology*.

[15] Haarmann A., Nowak E., Deiß A. (2015). Soluble VCAM-1 impairs human brain endothelial barrier integrity via integrin *α*-4-transduced outside-in signalling. *Acta Neuropathologica*.

[16] Sobhani A., Ebrahimi H., Ebrahimi A. (2018). VCAM-1 as a endothelial factor for diagnosis of dementia in Parkinson’s disease. *Joural of Neurology and Neuroscience*.

[17] Perner C., Perner F., Gaur N. (2019). Plasma VCAM1 levels correlate with disease severity in Parkinson’s disease. *Journal of Neuroinflammation*.

[18] Huang C.-W., Tsai M.-H., Chen N.-C. (2017). Clinical significance of circulating vascular cell adhesion molecule-1 to white matter disintegrity in Alzheimer's dementia. *Thrombosis and Haemostasis*.

[19] Yang Z., Wu L., Zhu X. (2013). MiR-29a modulates the angiogenic properties of human endothelial cells. *Biochemical and Biophysical Research Communications*.

[20] Serafin A., Foco L., Zanigni S. (2015). Overexpression of blood microRNAs 103a, 30b, and 29a in L-dopa-treated patients with PD. *Neurology*.

[21] Zlokovic B. V. (2008). The blood-brain barrier in health and chronic neurodegenerative disorders. *Neuron*.

[22] Hughes A. J., Ben-Shlomo Y., Daniel S. E., Lees A. J. (1992). What features improve the accuracy of clinical diagnosis in Parkinson's disease: a clinicopathologic study. *Neurology*.

[23] Folstein M. F., Folstein S. E., McHugh P. R. (1975). "Mini-mental state": A practical method for grading the cognitive state of patients for the clinician. *Journal of Psychiatric Research*.

[24] Hughes A. J., Daniel S. E., Kilford L., Lees A. J. (1992). Accuracy of clinical diagnosis of idiopathic Parkinson's disease: a clinico-pathological study of 100 cases. *Journal of Neurology, Neurosurgery, and Psychiatry*.

[25] Goetz C. G., Poewe W., Rascol O. (2004). Movement Disorder Society Task Force report on the Hoehn and Yahr staging scale: status and recommendations. *Movement Disorders*.

[26] Van Den Eeden S. K., Tanner C. M., Bernstein A. L. (2003). Incidence of Parkinson's disease: variation by age, gender, and race/ethnicity. *American Journal of Epidemiology*.

[27] Haaxma A., Bloem B. R., Borm G. F. (2007). Gender differences in Parkinson's disease. *Journal of Neurology, Neurosurgery, and Psychiatry*.

[28] Miller I. N., Cronin-Golomb A. (2010). Gender differences in Parkinson's disease: clinical characteristics and cognition. *Movement Disorders*.

[29] Ashburner J. (2007). A fast diffeomorphic image registration algorithm. *NeuroImage*.

[30] Sawada M., Imamura K., Nagatsu T. (2006). Role of cytokines in inflammatory process in Parkinson's disease. *Journal of Neural Transmission. Supplementum*.

[31] Pan P. L., Song W., Shang H. F. (2012). Voxel-wise meta-analysis of gray matter abnormalities in idiopathic Parkinson's disease. *European Journal of Neurology*.

[32] Chen F. X., Kang D. Z., Chen F. Y. (2016). Gray matter atrophy associated with mild cognitive impairment in Parkinson's disease. *Neuroscience Letters*.

[33] Tarawneh R., Head D., Allison S. (2015). Cerebrospinal fluid markers of neurodegeneration and rates of brain atrophy in early Alzheimer disease. *JAMA Neurology*.

[34] Lee S. Y., Chen M. H., Chiang P. L. (2018). Reduced gray matter volume and respiratory dysfunction in Parkinson's disease: a voxel-based morphometry study. *BMC Neurology*.

[35] Bodden M. E., Dodel R., Kalbe E. (2010). Theory of mind in Parkinson's disease and related basal ganglia disorders: a systematic review. *Movement Disorders*.

[36] Aminoff E. M., Kveraga K., Bar M. (2013). The role of the parahippocampal cortex in cognition. *Trends in Cognitive Sciences*.

[37] Sokolov A. A., Miall R. C., Ivry R. B. (2017). The cerebellum: adaptive prediction for movement and cognition. *Trends in Cognitive Sciences*.

[38] Pereira J. B., Junqué C., Martí M.-J., Ramirez-Ruiz B., Bargalló N., Tolosa E. (2009). Neuroanatomical substrate of visuospatial and visuoperceptual impairment in Parkinson's disease. *Movement Disorders*.

[39] Pagonabarraga J., Corcuera-Solano I., Vives-Gilabert Y. (2013). Pattern of regional cortical thinning associated with cognitive deterioration in Parkinson's disease. *PLoS One*.

[40] Xu Y., Yang J., Hu X., Shang H. (2016). Voxel-based meta-analysis of gray matter volume reductions associated with cognitive impairment in Parkinson's disease. *Journal of Neurology*.

[41] González-Redondo R., García-García D., Clavero P. (2014). Grey matter hypometabolism and atrophy in Parkinson’s disease with cognitive impairment: a two-step process. *Brain*.

[42] Lin W. C., Chen P. C., Huang Y. C. (2016). Dopaminergic therapy modulates cortical perfusion in Parkinson disease with and without dementia according to arterial spin labeled perfusion magnetic resonance imaging. *Medicine*.

[43] Nishio Y., Hirayama K., Takeda A. (2010). Corticolimbic gray matter loss in Parkinson's disease without dementia. *European Journal of Neurology*.

[44] Martin W. R. W., Wieler M., Gee M., Camicioli R. (2009). Temporal lobe changes in early, untreated Parkinson's disease. *Movement Disorders*.

[45] Rektor I., Svatkova A., Vojtisek L. (2018). White matter alterations in Parkinson's disease with normal cognition precede grey matter atrophy. *PLoS One*.

[46] Lin W. C., Chen H. L., Hsu T. W. (2017). Correlation between dopamine transporter degradation and striatocortical network alteration in Parkinson's disease. *Frontiers in Neurology*.

[47] Wise R. A. (2009). Roles for nigrostriatal--not just mesocorticolimbic--dopamine in reward and addiction. *Trends in Neurosciences*.

[48] Caminiti S. P., Presotto L., Baroncini D. (2017). Axonal damage and loss of connectivity in nigrostriatal and mesolimbic dopamine pathways in early Parkinson's disease. *Neuroimage Clin*.

[49] Zlokovic B. V. (2011). Neurovascular pathways to neurodegeneration in Alzheimer's disease and other disorders. *Nature Reviews. Neuroscience*.

[50] Stolp H. B., Dziegielewska K. M. (2009). Review: role of developmental inflammation and blood-brain barrier dysfunction in neurodevelopmental and neurodegenerative diseases. *Neuropathology and Applied Neurobiology*.

[51] Monahan A. J., Warren M., Carvey P. M. (2008). Neuroinflammation and peripheral immune infiltration in Parkinson's disease: an autoimmune hypothesis. *Cell Transplantation*.

[52] Daneman R., Prat A. (2015). The blood-brain barrier. *Cold Spring Harbor Perspectives in Biology*.

[53] Greenwood J., Heasman S. J., Alvarez J. I., Prat A., Lyck R., Engelhardt B. (2011). Review: leucocyte-endothelial cell crosstalk at the blood-brain barrier: a prerequisite for successful immune cell entry to the brain. *Neuropathology and Applied Neurobiology*.

[54] Allavena R., Noy S., Andrews M., Pullen N. (2010). CNS elevation of vascular and not mucosal addressin cell adhesion molecules in patients with multiple sclerosis. *The American Journal of Pathology*.

[55] Price D. T., Loscalzo J. (1999). Cellular adhesion molecules and atherogenesis. *The American Journal of Medicine*.

[56] Tchalla A. E., Wellenius G. A., Travison T. G. (2015). Circulating vascular cell adhesion molecule-1 is associated with cerebral blood flow dysregulation, mobility impairment, and falls in older adults. *Hypertension*.

[57] Novak V., Zhao P., Manor B. (2011). Adhesion molecules, altered vasoreactivity, and brain atrophy in type 2 diabetes. *Diabetes Care*.

[58] Purkayastha S., Fadar O., Mehregan A. (2013). Impaired cerebrovascular hemodynamics are associated with cerebral white matter damage. *Journal of Cerebral Blood Flow and Metabolism*.

[59] Barcia C. (2004). Blood vessels and parkinsonism. *Frontiers in Bioscience*.

[60] Janelidze S., Lindqvist D., Francardo V. (2015). Increased CSF biomarkers of angiogenesis in Parkinson disease. *Neurology*.

[61] Gu W., Zhan H., Zhou X. Y. (2017). MicroRNA-22 regulates inflammation and angiogenesis via targeting VE-cadherin. *FEBS Letters*.

[62] Wang J., Wang Y., Wang Y., Ma Y., Lan Y., Yang X. (2013). Transforming growth factor *β*-regulated microRNA-29a promotes angiogenesis through targeting the phosphatase and tensin homolog in endothelium. *The Journal of Biological Chemistry*.

[63] Roshan R., Shridhar S., Sarangdhar M. A. (2014). Brain-specific knockdown of miR-29 results in neuronal cell death and ataxia in mice. *RNA*.

[64] Liu C. Z., Zhong Q., Huang Y. Q. (2017). Elevated plasma miR-29a levels are associated with increased carotid intima-media thickness in atherosclerosis patients. *The Tohoku Journal of Experimental Medicine*.

[65] Haas S. J.-P., Zhou X., Machado V., Wree A., Krieglstein K., Spittau B. (2016). Expression of Tgf*β*1 and inflammatory markers in the 6-hydroxydopamine mouse model of Parkinson's disease. *Frontiers in Molecular Neuroscience*.

[66] Margis R., Margis R., Rieder C. R. M. (2011). Identification of blood microRNAs associated to Parkinsońs disease. *Journal of Biotechnology*.

[67] Muller M., Jakel L., Bruinsma I. B., Claassen J. A., Kuiperij H. B., Verbeek M. M. (2016). MicroRNA-29a is a candidate biomarker for Alzheimer's disease in cell-free cerebrospinal fluid. *Molecular Neurobiology*.

[68] Shioya M., Obayashi S., Tabunoki H. (2010). Aberrant microRNA expression in the brains of neurodegenerative diseases: miR-29a decreased in Alzheimer disease brains targets neurone navigator 3. *Neuropathol Appl Neurobiol*.

[69] Price C. F., Burgess D. J., Kastellorizios M. (2016). l-DOPA as a small molecule surrogate to promote angiogenesis and prevent dexamethasone-induced ischemia. *Journal of Controlled Release*.

[70] Chen H. L., Lin H. C., Lu C. H. (2017). Systemic inflammation and alterations to cerebral blood flow in obstructive sleep apnea. *Journal of Sleep Research*.

[71] Jellema R. K., Lima Passos V., Zwanenburg A. (2013). Cerebral inflammation and mobilization of the peripheral immune system following global hypoxia-ischemia in preterm sheep. *Journal of Neuroinflammation*.

[72] Chen Y. S., Chen H. L., Lu C. H. (2019). Reduced lateral occipital gray matter volume is associated with physical frailty and cognitive impairment in Parkinson's disease. *European Radiology*.

[73] Chiang P. L., Chen H. L., Lu C. H. (2018). Interaction of systemic oxidative stress and mesial temporal network degeneration in Parkinson's disease with and without cognitive impairment. *Journal of Neuroinflammation*.

[74] Grill-Spector K., Weiner K. S. (2014). The functional architecture of the ventral temporal cortex and its role in categorization. *Nature Reviews. Neuroscience*.

[75] Christopher L., Duff-Canning S., Koshimori Y. (2015). Salience network and parahippocampal dopamine dysfunction in memory-impaired Parkinson disease. *Annals of Neurology*.

[76] Ward A. M., Schultz A. P., Huijbers W., van Dijk K. R. A., Hedden T., Sperling R. A. (2014). The parahippocampal gyrus links the default-mode cortical network with the medial temporal lobe memory system. *Human Brain Mapping*.

[77] Swirski M., Miners J. S., de Silva R. (2014). Evaluating the relationship between amyloid-*β* and *α*-synuclein phosphorylated at Ser129 in dementia with Lewy bodies and Parkinson's disease. *Alzheimer's Research & Therapy*.

[78] Guan J., Pavlovic D., Dalkie N. (2013). Vascular degeneration in Parkinson's disease. *Brain Pathology*.

[79] Bennett R. E., Robbins A. B., Hu M. (2018). Tau induces blood vessel abnormalities and angiogenesis-related gene expression in P301L transgenic mice and human Alzheimer's disease. *Proc Natl Acad Sci U S A*.

[80] Sweeney M. D., Sagare A. P., Zlokovic B. V. (2018). Blood-brain barrier breakdown in Alzheimer disease and other neurodegenerative disorders. *Nature Reviews. Neurology*.

[81] Brysbaert M. (2019). How many participants do we have to include in properly powered experiments? A Tutorial of Power Analysis with Reference Tables. *Journal of Cognition*.

